# Bilateral lung transplantation with simultaneous aortic replacement using donor aorta in a patient with pulmonary arterial hypertension and patent ductus arteriosus

**DOI:** 10.1186/s13019-025-03808-w

**Published:** 2026-01-03

**Authors:** Naoya Ishida, Hisashi Oishi, Hiromichi Niikawa, Takaya Suzuki, Hirotsugu Notsuda, Takashi Hirama, Tatsuaki Watanabe, Yui Watanabe, Takeo Togo, Ken Onodera, Sakiko Kumata, Yuyo Suzuki, Takayasu Ito, Ryuichi Taketomi, Shintaro Katahira, Kiichiro Kumagai, Yoshikatsu Saiki, Yoshinori Okada

**Affiliations:** 1 Department of Thoracic Surgery, Institute of Development, Aging and Cancer, Tohoku University, 4 − 1 Seiryocho, Aoba-ku, Sendai, 980–8575 Japan; 2 Division of Cardiovascular Surgery, Tohoku University Graduate School of Medicine, 1–1 Seiryocho, Aoba-ku, Sendai, 980–8575 Japan

**Keywords:** Aortic replacement, Congenital heart disease, Lung transplantation, Patent ductus arteriosus, Pulmonary artery aneurysm, Pulmonary arterial hypertension

## Abstract

**Background:**

A patent ductus arteriosus (PDA) can result in pulmonary arterial hypertension (PAH) due to a left-to-right shunt. Lung transplantation (LTx) is indicated when PAH becomes refractory to medical management. We report a case of bilateral LTx (BLTx) with simultaneous aortic replacement using a donor aorta in an adult patient with PAH complicated by PDA.

**Case presentation:**

A 27-year-old woman was referred for an LTx evaluation. At 1-year-old, she was diagnosed with a PDA. At the time of diagnosis, PDA closure was not indicated due to severe PAH, with a pulmonary vascular resistance of 33.8 Wood units. Despite receiving maximal medical therapy, her condition progressively deteriorated. She was placed on the transplant waitlist at age 27. Since left ventricular function was preserved (ejection fraction 60%) and no complex congenital heart disease was present, bilateral lung transplantation was chosen instead of heart-lung transplantation. Preoperative computed tomography revealed a giant pulmonary artery aneurysm (PAA). At 31 years of age, she underwent BLTx with simultaneous replacement of the proximal descending thoracic aorta using a donor aortic graft under cardiopulmonary bypass to enable complete excision of the ductal tissue. The giant PAA was also repaired during the same procedure. Postoperatively, she required venoarterial extracorporeal membrane oxygenation and was successfully weaned off by postoperative day 4. After an extended rehabilitation period, the patient was discharged 153 days postoperatively and remained in good health for 16 months following transplantation.

**Conclusions:**

To our knowledge, this is the first reported case of LTx with aortic replacement using a donor aortic graft for the management of PDA. We believe this combined procedure may represent a feasible surgical strategy for adult patients with PAH complicated by PDA and warrants further investigation in future cases.

## Background

Lung transplantation (LTx) remains the definitive treatment for patients with severe, medication-resistant pulmonary arterial hypertension (PAH). In cases of PAH associated with congenital heart disease, LTx with simultaneous intracardiac repair of associated defects, such as atrial septal defect or patent ductus arteriosus (PDA), is indicated.

Here, we describe a novel surgical approach for treating an adult patient with PAH and PDA: bilateral lung transplantation (BLTx) combined with aortic replacement using a donor aorta.

## Case presentation

**Fig. 1:**
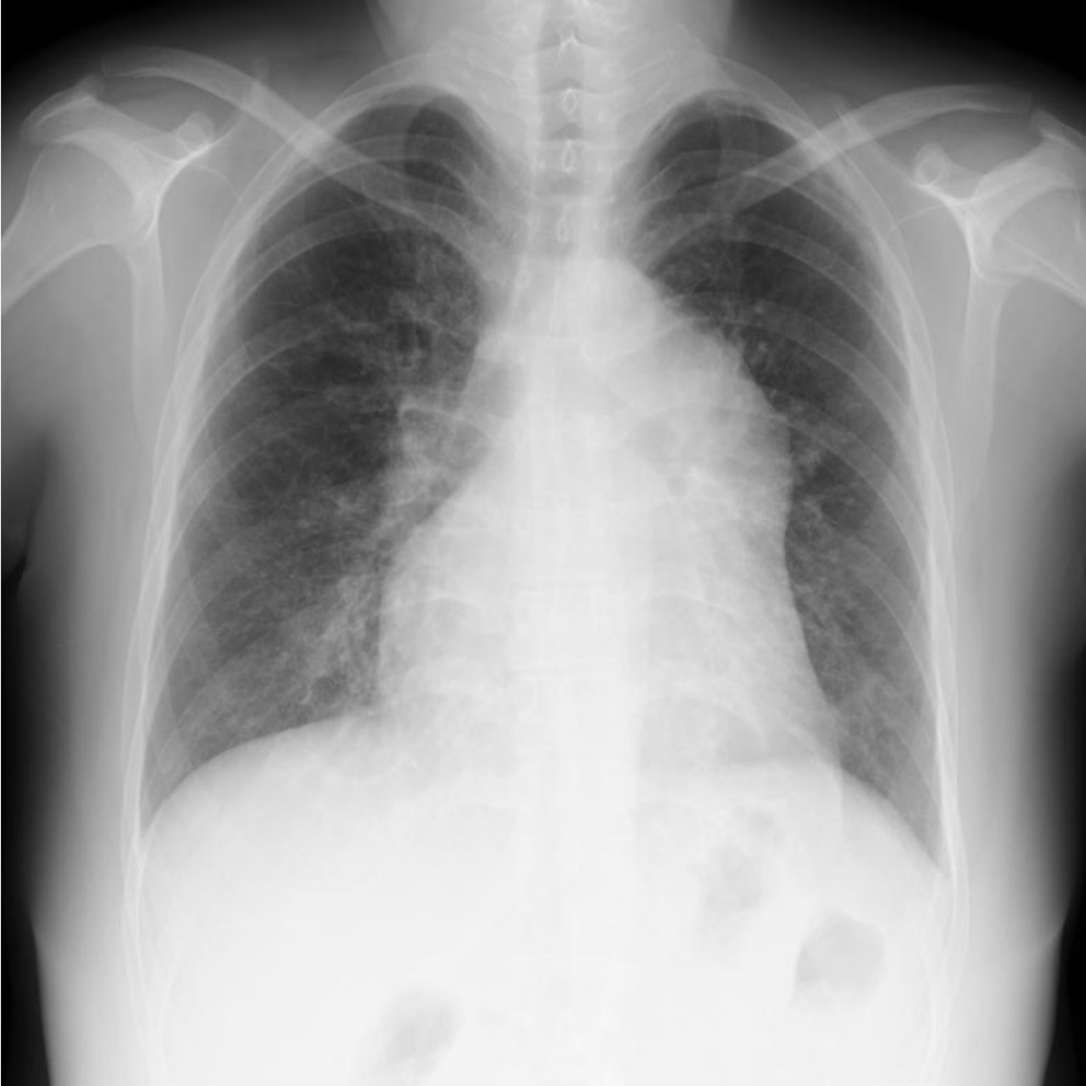
Preoperative chest radiograph. The image shows an enlarged mediastinal shadow

**Fig. 2:**
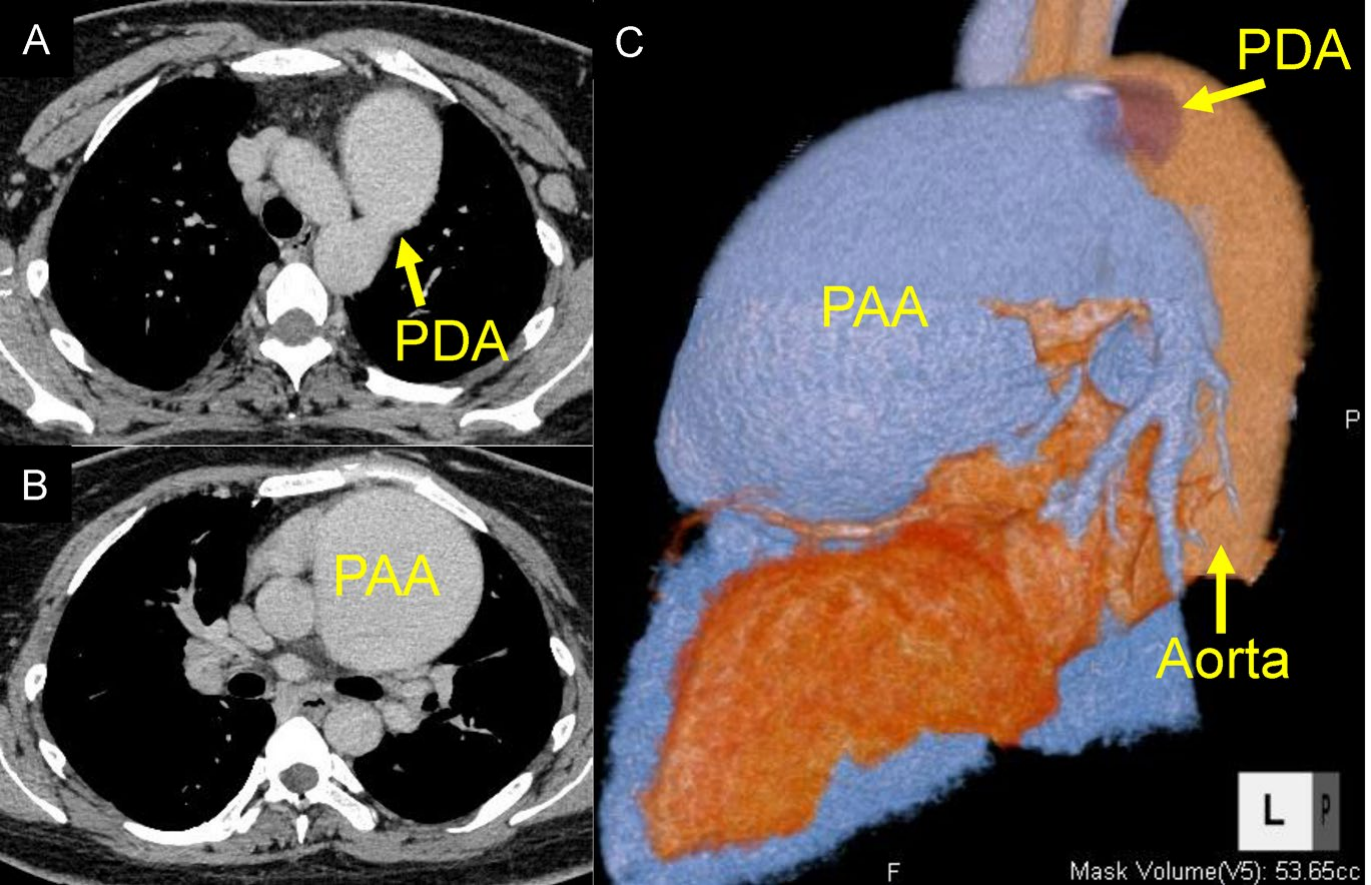
Preoperative chest computed tomography (CT) and three-dimensional reconstructed CT. (**A**) Chest CT showing a patent ductus arteriosus (PDA) with an internal diameter of 15 mm. (**B**) Chest CT revealing a giant pulmonary artery aneurysm (PAA) measuring up to 8 cm in the pulmonary artery trunk. (**C**) Three-dimensional reconstructed CT highlighting the large PAA and PDA in relation to the aorta

A 27-year-old woman was referred for an LTx evaluation. She had been diagnosed with a PDA at the age of one. At that time, PDA closure was not indicated due to the presence of severe PAH, with a pulmonary vascular resistance of 33.8 Wood units. The patient was treated with endothelin receptor antagonists and phosphodiesterase type 5 inhibitors. Despite these interventions, her condition progressively worsened. By age 23, she had reached World Health Organization (WHO) functional class IV and exhibited differential cyanosis between the upper and lower extremities, consistent with a right-to-left shunt. Her Qp/Qs ratio was 0.6. As her condition continued to decline despite maximal medical therapy, including phosphodiesterase type 5 inhibitors, endothelin receptor antagonists, prostacyclin analogs, and prostacyclin receptor agonists, she was placed on the transplant waitlist at age 27. At the time of listing, she was wheelchair-dependent. Chest radiography revealed an enlarged mediastinal shadow (Fig. 1). Computed tomography (CT) revealed a PDA with an internal diameter of 15 mm and a pulmonary artery aneurysm (PAA) measuring up to 80 mm in the pulmonary artery trunk (Fig. 2). Echocardiography demonstrated impaired right ventricular function with a fractional area change of 28.7%, while left ventricular function was preserved, with an ejection fraction of 60%. Right heart catheterization revealed severe pulmonary hypertension (125/80 mmHg) and moderate pulmonary regurgitation.

**Fig. 3:**
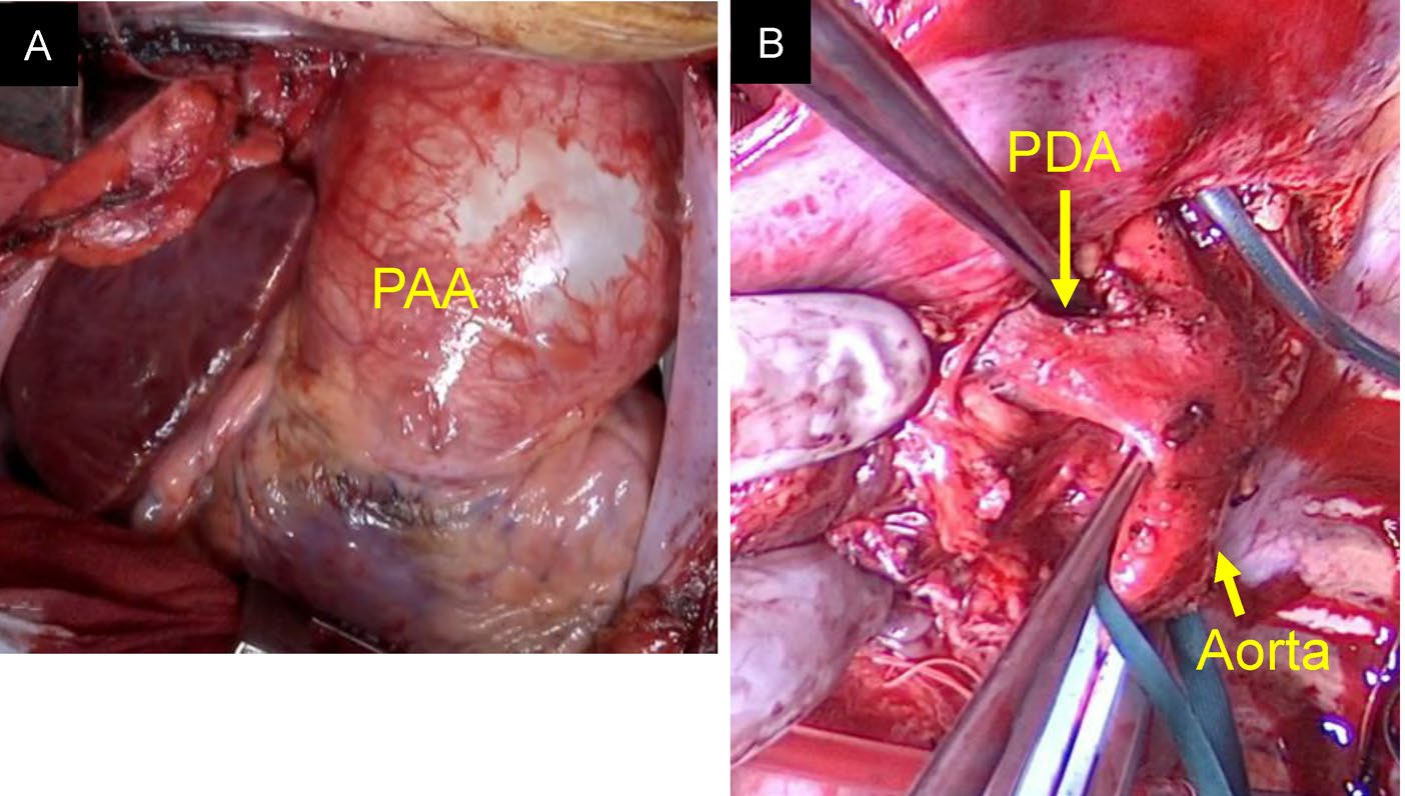
Intraoperative Images of the pulmonary artery trunk and the PDA. (**A**) Photograph taken immediately after opening the chest, showing a giant pulmonary artery aneurysm (PAA) involving the pulmonary artery trunk. (**B**) Image showing the large arterial duct (PDA) prior to dissection

**Fig. 4:**
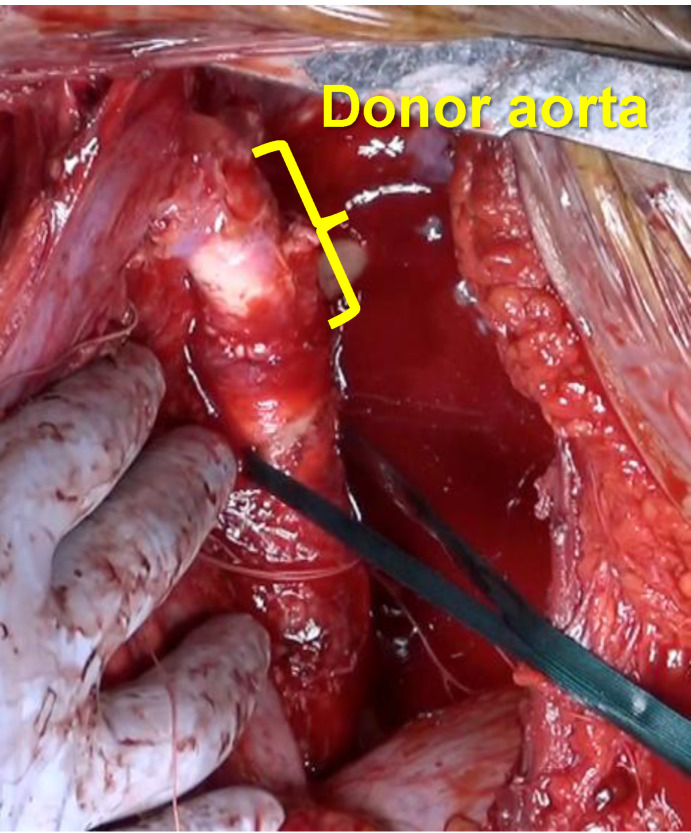
Image showing the descending thoracic aorta after aortic replacement using a donor aorta. Approximately 30 mm of the proximal descending thoracic aorta, specifically the segment surrounding the aortic orifice of the PDA, was resected and replaced with a graft fashioned from the donor descending thoracic aorta using continuous running sutures

**Fig. 5:**
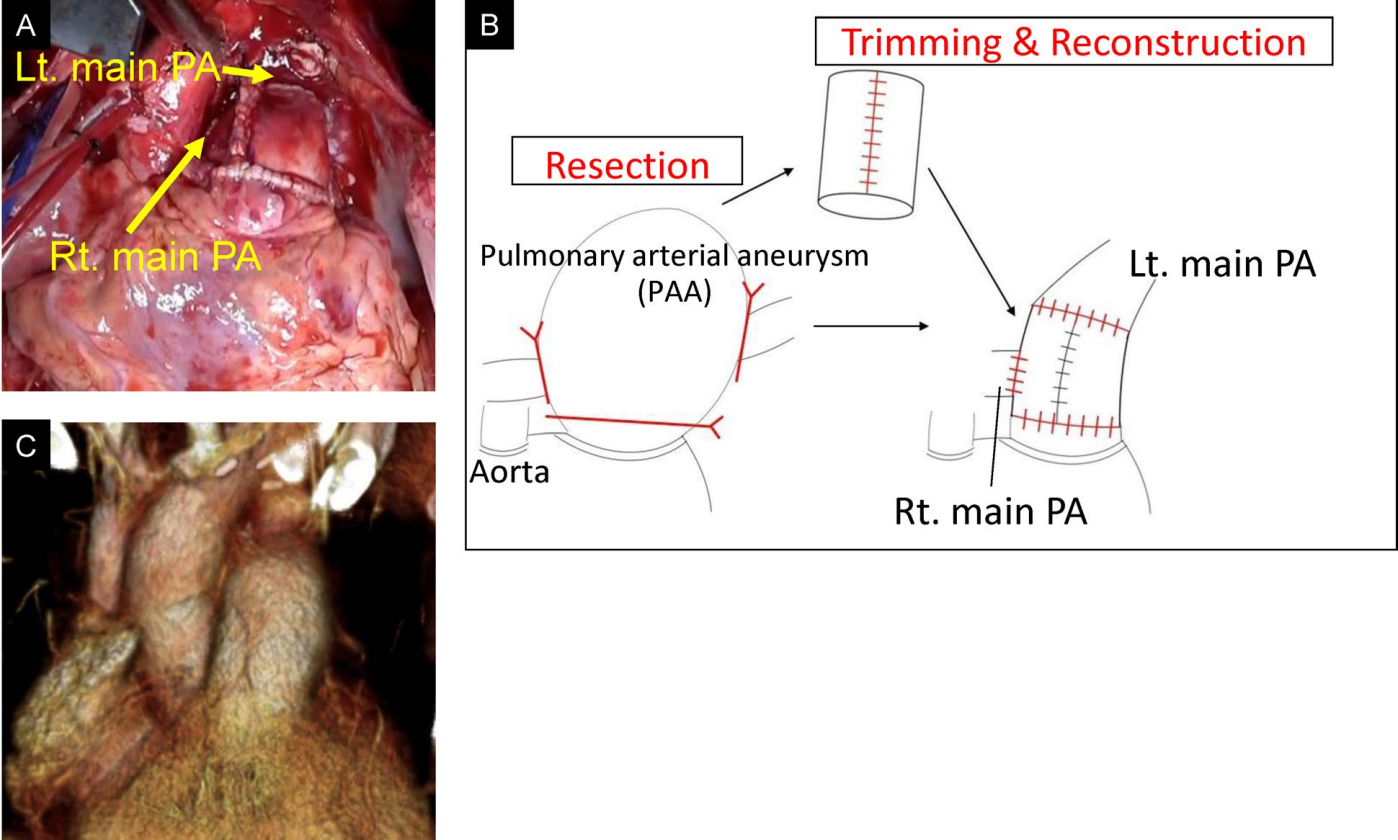
Images of the pulmonary artery trunk after reconstruction. (**A**) Photograph after completion of pulmonary artery trunk reconstruction and all anastomoses. (**B**) Schematic illustration of the reconstruction technique. The aneurysmal pulmonary trunk was resected and replaced with a new cylindrical conduit approximately 30 mm in diameter. The recreated pulmonary trunk was connected to the left main pulmonary artery (Lt. main PA) by an end-to-end anastomosis, while the right main pulmonary artery (Rt. main PA) was anastomosed to the pulmonary trunk in an end-to-side fashion. The proximal end of the pulmonary trunk was anastomosed to the pulmonary sinotubular junction. (**C**) Three-dimensional reconstructed CT showing a new pulmonary artery trunk. The image was obtained approximately one year after transplantation, demonstrating stability of the reconstructed conduit without aneurysmal recurrence

**Fig. 6:**
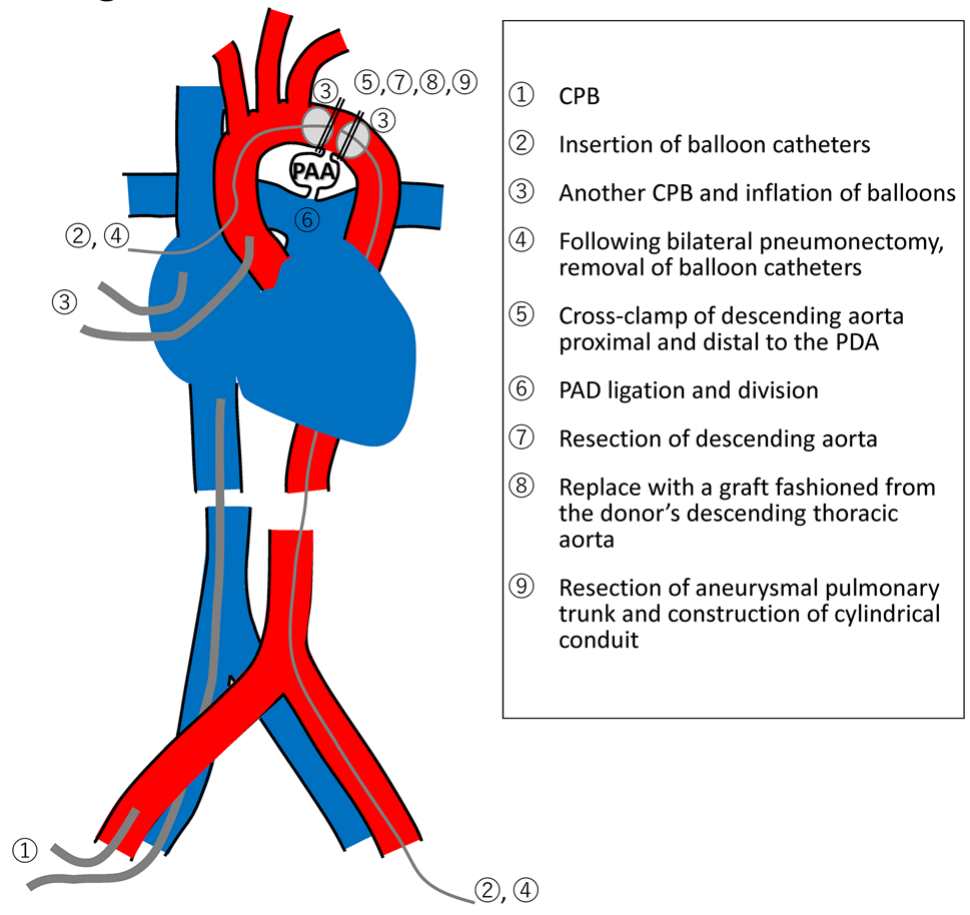
Schematic diagram of the operative sequence and cardiopulmonary bypass (CPB) cannulation strategy. CPB was initiated with right atrial drainage via the right femoral vein and arterial return through the right femoral artery. Additional cannulas were inserted into the ascending aorta and superior vena cava to maintain cerebral and upper-body perfusion during clamping of the distal aortic arch for aortic replacement. Balloon catheters were introduced from the ascending aorta (proximal to the PDA) and from the left femoral artery (distal to the PDA) to control blood flow across the ductus and prevent left-ventricular overdistention. These catheters were positioned before lung explantation and removed after bilateral pneumonectomy. Aortic replacement was performed first, followed by reconstruction of the pulmonary artery trunk and then bilateral lung implantation. PAA, pulmonary artery aneurysm. PDA, patent ductus arteriosus

After a 4-year wait on the transplant list, the patient underwent BLTx. The donor was a 44-year-old woman with no history of smoking and an arterial oxygen tension/inspired oxygen fraction (PaO_2_/FiO_2_) ratio of > 500, indicating excellent graft quality. The recipient’s blood type was B, and the donor’s blood type was compatible. The predicted forced vital capacity (pFVC) size difference between donor and recipient was − 9.3%, which was considered acceptable for bilateral lung transplantation. HLA matching revealed shared alleles for B52, Cw7, Cw12, DR15, and DQ6. The donor’s lungs showed no signs of atelectasis or pneumonia. General anesthesia was induced, and the chest was accessed via a clamshell incision. Intraoperative monitoring included transesophageal echocardiography (TEE) and Swan-Ganz catheterization. Upon opening the pericardium, a giant PAA was immediately identified (Fig. 3 A). Cardiopulmonary bypass (CPB) was initiated with right atrial drainage via the right femoral vein and arterial return through the right femoral artery. A balloon catheter was inserted through the ascending aorta toward the proximal aortic orifice of the PDA, and another catheter was inserted via the left femoral artery toward the distal PDA orifice. Additional CPB circuits were established through the ascending aorta and superior vena cava to maintain cerebral and upper body perfusion during subsequent clamping of the distal aortic arch for the aortic replacement procedure. The balloons were inflated to prevent left ventricular overdistention. These catheters were placed before lung explantation to minimize manipulation near the PDA. Balloon positions were confirmed using intraoperative fluoroscopy and TEE. Following bilateral pneumonectomy, the balloon catheters were removed, and the descending aorta was cross-clamped proximal and distal to the PDA. The PDA was then dissected, ligated, and divided (Fig. 3B). Approximately 30 mm of the descending aorta was resected and replaced with a graft fashioned from the donor’s descending thoracic aorta surrounding the aortic orifice of the PDA using continuous suturing (Fig. 4). Due to thinning of the recipient’s aortic wall, an autologous pericardial strip was applied to reinforce the proximal anastomosis. The aneurysmal pulmonary trunk was resected, and a cylindrical conduit approximately 30 mm in diameter was constructed using the macroscopically preserved portion of the recipient’s native pulmonary artery wall. Central plication was performed on each pulmonary valve cusp to improve coaptation depth. The reconstructed pulmonary trunk was anastomosed to the left main pulmonary artery in an end-to-end fashion and to the right main pulmonary artery in an end-to-side configuration. The proximal end of the new pulmonary trunk was then connected to the pulmonary sinotubular junction (Fig. 5 A, B, C). Following vascular reconstruction, donor lung grafts were transplanted. Due to the complexity of the procedure, ischemic times were 10 h and 18 min for the first lung and 12 h and 45 min for the second lung. A schematic summarizing the operative sequence and cannulation strategy from CPB to resection of aneurysmal pulmonary trunk and construction of cylindrical conduit is shown in Fig. 6.

**Fig. 7:**
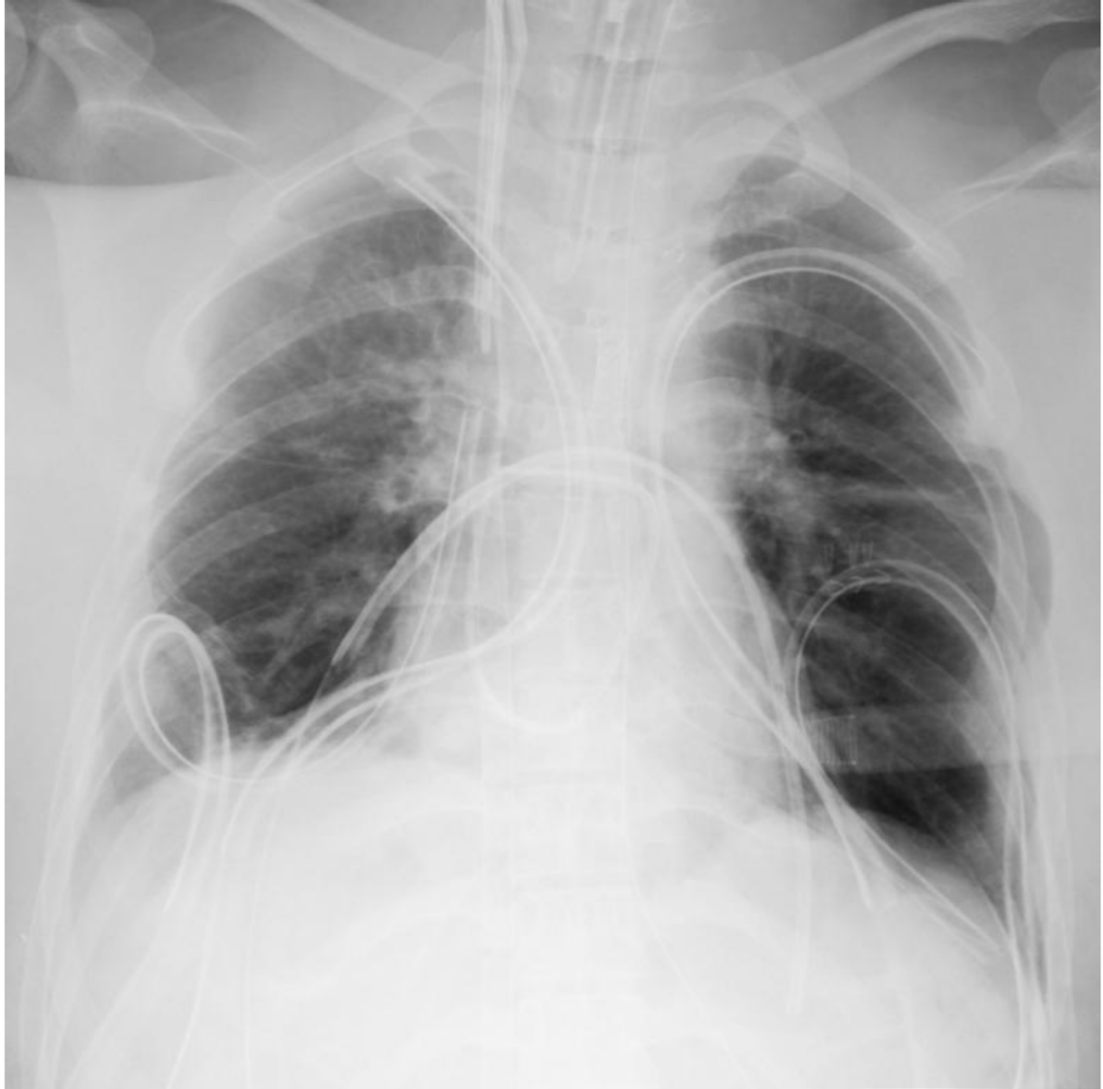
Postoperative chest radiography. A chest radiograph obtained after LTx showing ground-glass opacity in the right upper lung field

During the attempt to wean from CPB, elevated pulmonary vascular resistance and signs of left and right heart dysfunction were observed. Intraoperative left ventricular failure was considered a major factor in the need for VA-ECMO, although the prolonged ischemic times may also have contributed. CPB was converted to central venoarterial extracorporeal membrane oxygenation (VA-ECMO), and the patient was transferred to the intensive care unit (ICU). Postoperative chest radiography revealed ground-glass opacity in the right upper lung field, and the patient was classified as grade 3 primary graft dysfunction (Fig. 7). On postoperative day (POD) 4, support was transitioned from VA-ECMO to venovenous extracorporeal membrane oxygenation (VV-ECMO) via bilateral femoral vein cannulation, and the chest was closed. Right heart catheterization data obtained after VA-ECMO withdrawal showed a pulmonary artery pressure of 22/7 mmHg (mean 14), central venous pressure (CVP) of 9 mmHg, cardiac output (CO) of 5.80 L/min, and cardiac index (CI) of 3.31 L/min/m². Transthoracic echocardiography demonstrated preserved right ventricular systolic function, and no significant pulmonary valve dysfunction was observed apart from mild regurgitation. VV-ECMO was successfully weaned on POD 8, and ventilator support was discontinued on POD 9. The patient was discharged from the ICU on POD 26. Postoperative transthoracic echocardiography revealed mild pulmonary valve regurgitation. The tricuspid regurgitation pressure gradient (TRPG) decreased significantly from 98 mmHg preoperatively to 23 mmHg postoperatively, indicating substantial hemodynamic improvement. The patient developed postoperatively chylothorax, which initially failed to resolve with pleurodesis but was successfully treated with thoracic duct ligation on POD 87. Given her preoperative physical deconditioning, she required prolonged rehabilitation. She was discharged on POD 153 and remained in good health 18 months following transplantation. Postoperative immunosuppression consisted of basiliximab induction, tacrolimus (target trough levels: 10–14 ng/mL during the first 6 months), mycophenolate mofetil (1000–1500 mg depending on body weight), and corticosteroids tapered to maintenance doses. Prophylactic antimicrobials included piperacillin/tazobactam or alternatives until chest tubes were removed, micafungin followed by voriconazole for antifungal coverage, valganciclovir for CMV prophylaxis, and lifelong trimethoprim-sulfamethoxazole for Pneumocystis prophylaxis.

### Discussion and conclusions

There are few reports of LTx combined with PDA closure in adult patient. In previously documented cases, the PDA was closed using conventional surgical ligation or catheter-based techniques [1]. To the best of our knowledge, this is the first reported case of LTx with simultaneous aortic replacement using a donor aortic graft for PDA management.

Approaches to PDA closure vary between pediatric and adult patients. In pediatric cases, standard techniques include surgical ligation or division of the ductus. In adult patients, however, ligation is more technically challenging due to the increased fragility of the ductal wall and extensive periductal calcification [2]. Endovascular or patch closure of the pulmonary orifice of the ductus is more commonly employed in adults [3, 4]; However, endovascular approaches are not suitable for large-diameter PDA lesions [5]. Moreover, aneurysm formation has been reported in residual ductal tissue following patch closure at the pulmonary orifice [6, 7]. Given these considerations, we elected to replace the descending thoracic aorta with a donor aortic graft that included the PDA orifice. A previous report described the successful treatment of an aneurysm that developed on the lesser curvature of the aortic arch after PDA surgery using a custom-made novel stent graft in an adult patient with residual PDA tissue [8]. Hybrid endovascular–surgical approach which might have been applicable in our case. In addition, there are case reports of large PDA closure without aortic replacement, although these often involve smaller ductal orifices or less extensive tissue involvement. In contrast, our patient had a markedly dilated PDA (15 mm aortic orifice) with associated aneurysmal changes, prompting complete excision and replacement with donor aorta to ensure durability. In this case, the aortic opening of the PDA was approximately 15 mm in diameter, which we considered unusually large. Simple ligation and division, or patch closure at the pulmonary artery side, might have left residual ductal tissue at the aortic wall, posing a potential risk for postoperative aneurysm formation. Therefore, complete excision of the involved aortic segment was performed.

Use of an artificial vascular graft was avoided due to the need for high-dose immunosuppression following transplantation. We considered that the use of donor aorta would reduce the risk of graft-related infection compared to synthetic materials. While donor aorta was selected to reduce early graft-related infection, we acknowledge that this approach carries potential long-term risks, including degeneration, dilatation, and graft infection. Therefore, lifelong imaging surveillance is essential to monitor graft integrity and detect any delayed complications. Follow-up surveillance includes contrast-enhanced computed tomography (CT) performed at regular intervals, with the most recent scan at one year showing no evidence of aneurysmal recurrence. Long-term imaging follow-up is ongoing to monitor for late complications such as graft degeneration, infection, or dilatation.

Reconstruction of the pulmonary artery trunk presents additional surgical challenges. In this case, the donor heart was allocated to another recipient, and therefore the donor pulmonary artery trunk was not available for reconstruction. The aneurysmal pulmonary trunk was resected, and a new conduit was fashioned from the macroscopically preserved portion of the recipient’s native pulmonary arterial wall. Yokoyama et al. reported a case of living-donor lobar lung transplantation for PAH with severe pulmonary artery dilation. In that case, the dilated trunk was managed by resecting the anterior wall and directly suturing the remaining pulmonary artery wall [8]. In our patient, the pulmonary sinotubular junction was markedly dilated, and simple plication of the pulmonary trunk was expected to distort the pulmonary valve. Therefore, we elected to perform a complete reconstruction of the pulmonary trunk, followed by central plication of the pulmonary valve cusps.

Due to the complexity of the procedures, ischemic time was prolonged. Although anesthesia was initiated in the recipient 2 h before donor surgery, the ischemic time of the second lung reached 12 h and 45 min. Extracellular phosphate-buffered lung preservation solution (EP-TU solution) was used, as it has been reported to support prolonged graft viability [9]. In Japan, 10 °C preservation has not yet been introduced into clinical practice, and while a few case reports of ex vivo lung perfusion (EVLP) exist, EVLP is not routinely employed in most centers, including ours. Nonetheless, graft preservation has inherent time limitation. The patient required VA-ECMO for 4 days, followed by VV-ECMO for an additional 4 days. This case underscores the critical importance of optimal donor selection and precise coordination between thoracic and cardiovascular surgical teams when LTx is combined with complex adjunctive procedures.

The prolonged ischemic time in this case was primarily due to the surgical sequence, in which the donor aortic graft and lung grafts arrived simultaneously, and aortic replacement was prioritized before lung implantation. This likely contributed to the development of grade-3 primary graft dysfunction.

In conclusion, BLTx with simultaneous aortic replacement using a donor aorta may be a feasible surgical strategy for selected adult patients with PAH and PDA. This case highlights the importance of comprehensive preoperative planning and interdisciplinary collaboration. Further clinical experience is needed to assess the safety, reproducibility, and long-term outcomes of this approach.

## Data Availability

Not applicable.

## Abbreviations

CPB: Cardiopulmonary bypass
CT: Computed tomography
BLTx: Bilateral lung transplantation
ICU: Intensive care unit
LTx: Lung transplantation
PAA: Pulmonary artery aneurysm
PAH: Pulmonary arterial hypertension
PDA: Patent ductus arteriosus
POD: Post operative day
VA-ECMO: veno-arterial extracorporeal membrane oxygenation
VV-ECMO: veno-venous extracorporeal membrane oxygenation

## Declarations

### Use of large Language models (LLMs)

During the manuscript preparation, the authors used Chat GPT 4 for English language editing. The content was subsequently reviewed and revised by the authors, who take full responsibility for the final version. Additional English editing was performed by a native English speaker.
